# 4-Bromo-*N*-(4-hy­droxy­benzyl­idene)­aniline

**DOI:** 10.1107/S1600536812006101

**Published:** 2012-02-17

**Authors:** L. Jothi, G. Vasuki, R. Ramesh Babu, K. Ramamurthi

**Affiliations:** a Department of Physics, NKR Government Arts College for Women, Namakkal-1, India; b Department of Physics, Kunthavai Naachiar Government Arts College (W) (Autonomous), Thanjavur-7, India; c Crystal Growth and Thin Film Laboratory, School of Physics, Bharathidasan University, Tiruchirappalli-24, India

## Abstract

In the title compound, C_13_H_10_BrNO, the benzene ring planes are inclined at an angle of 48.85 (17)°, resulting in a nonplanar mol­ecule. A characteristic of aromatic Schiff bases with *N*-aryl substituents is that the terminal phenyl rings are twisted relative to the HC=N plane. In this case, the HC=N unit makes dihedral angles of 11.1 (4) and 38.5 (3)° with the hy­droxy­benzene and bromo­benzene rings, respectively. In the crystal, the molecules are linked by O—H⋯N hydrogen bonds to form infinite (*C*8) chains along the *b* axis.

## Related literature

For applications of Schiff base compounds and related structures, see: Li *et al.* (2008 ▶); Zhang (2010 ▶). For other related structures, see: Kaitner & Pavlovic (1995 ▶); Yeap *et al.* (1993 ▶). For an early determination of the lattice parameters of this compound, see: Bürgi *et al.* (1968 ▶). For standard bond lengths, see: Allen *et al.* (1987 ▶).
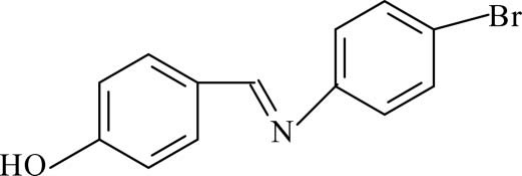


## Experimental

### 

#### Crystal data

C_13_H_10_BrNO
*M* *_r_* = 276.13
Orthorhombic, 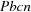

*a* = 21.9588 (10) Å
*b* = 11.0866 (5) Å
*c* = 9.3132 (4) Å
*V* = 2267.28 (17) Å^3^
*Z* = 8
Mo *K*α radiation
μ = 3.60 mm^−1^
*T* = 293 K
0.30 × 0.20 × 0.20 mm

#### Data collection

Bruker Kappa APEXII CCD diffractometer
Absorption correction: multi-scan (*SADABS*; Bruker, 2004 ▶) *T*_min_ = 0.452, *T*_max_ = 0.571
20366 measured reflections
2001 independent reflections
1494 reflections with *I* > 2σ(*I*)
*R*_int_ = 0.046

#### Refinement

*R*[*F* ^2^ > 2σ(*F* ^2^)] = 0.056
*wR*(*F* ^2^) = 0.174
*S* = 1.03
2001 reflections
145 parameters
H-atom parameters constrained
Δρ_max_ = 1.32 e Å^−3^
Δρ_min_ = −1.62 e Å^−3^

### 

Data collection: *APEX2* (Bruker, 2004 ▶); cell refinement: *APEX2* and *SAINT* (Bruker, 2004 ▶); data reduction: *SAINT* and *XPREP* (Bruker, 2004 ▶); program(s) used to solve structure: *SIR92* (Altomare *et al.*, 1994 ▶); program(s) used to refine structure: *SHELXL97* (Sheldrick, 2008 ▶); molecular graphics: *ORTEP-3* (Farrugia, 1997 ▶) and *PLATON* (Spek, 2009 ▶); software used to prepare material for publication: *SHELXL97*.

## Supplementary Material

Crystal structure: contains datablock(s) I, global. DOI: 10.1107/S1600536812006101/sj5193sup1.cif

Structure factors: contains datablock(s) I. DOI: 10.1107/S1600536812006101/sj5193Isup2.hkl

Supplementary material file. DOI: 10.1107/S1600536812006101/sj5193Isup3.cml

Additional supplementary materials:  crystallographic information; 3D view; checkCIF report

## Figures and Tables

**Table 1:** Hydrogen-bond geometry (Å, °)

*D*—H⋯*A*	*D*—H	H⋯*A*	*D*⋯*A*	*D*—H⋯*A*
O1—H1⋯N1^i^	0.82	1.92	2.734	175

Symmetry code: (i) 
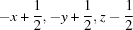
.

**Fig. 1.**
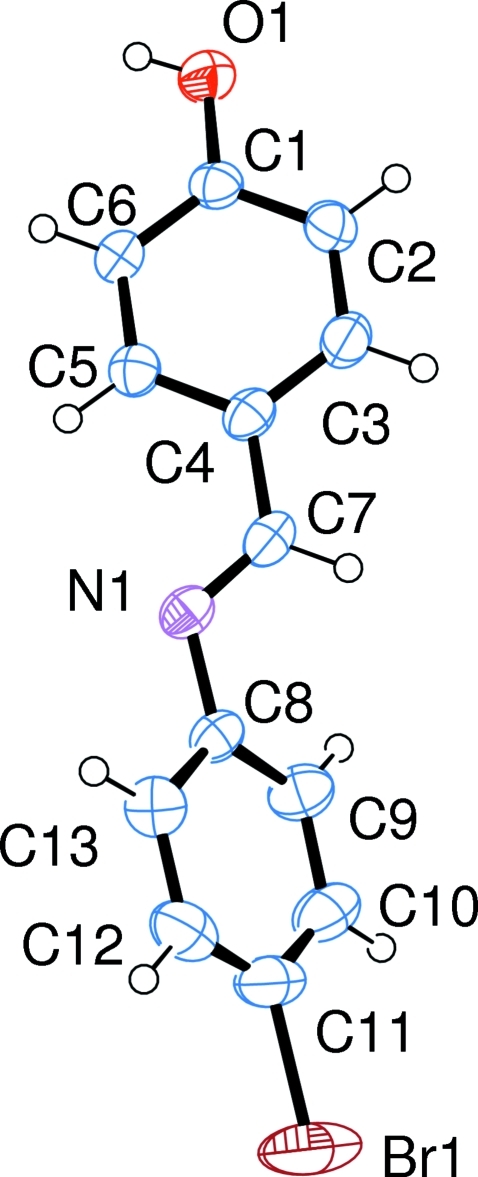
The molecular structure of the title compound, with atom numbering and displacement ellipsoids drawn at the 50% probability level.

**Fig. 2.**
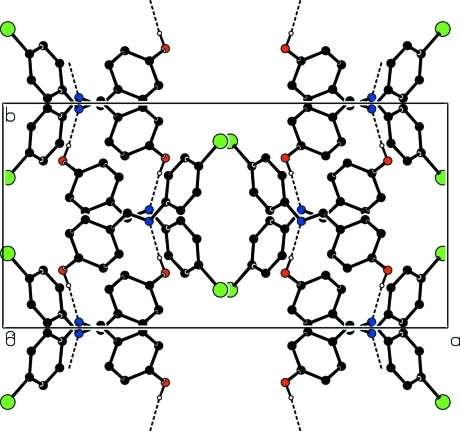
Crystal packing of the title compound viewed down the c axis showing O–H···N interactions as dashed lines (see Table 1 for details).

## supplementary crystallographic information

### Comment

Schiff base compounds have been used as fine chemicals and medical substrates. They are important ligands in co-ordination chemistry due to their ease of preparation and their ability to be modified both electronically and sterically (Li *et al.*, 2008 and Zhang, 2010). As a part of our study on the co-ordination behaviour of a ligand having a 4-hydroxy substituent on the benzylidene fragment, *X*- ray structural analysis of the title compound was carried out, the results are reported herein. The lattice parameters of this compound, determined from precession photographs, were reported previously by Bürgi *et al.* (1968). The title compound, (I), contains two benzene rings bridged by a C ═N imino moiety, the planes of which are inclined at an angle of 48.85 (17)°, showing significant deviation of the molecule from planarity as observed in a related structure *N*-p-tolylvanillaldimine (Kaitner & Pavlovic, 1995). The molecule exists in the solid state in an *E*-Configuration with respect to the C7═N1 bond as indicated by the torsion angle C4–C7–N1–C8 = 171.22 (4)°. In order to minimize the interaction between the hydroxy proton and H6 at C6 the O1–C1–C6 angle [123.4 (4)°] is larger than the O1–C1–C2 angle [117.4 (4)°] (Yeap *et al.*, 1993). The N1–C7–C4 [124.70 (4)°] is greater than the normal value of 120°; this might be a consequence of repulsion between the lone pair of electrons on N1 and H5 attached to C5 (N1···H5 = 2.6583 (1) Å). The C4-C7 [1.454 (6)Å] and N1-C8 [1.412 (6)Å] distances confirm a degree of π-electron delocalization between the benzene rings, and the molecule can be regarded as a partially delocalized π-electron system as observed in the related structures 4-[(3-methoxyphenylimino)methyl]phenol) and *N*-p-tolylvanillaldimine (Yeap, *et al.,*, 1993; Kaitner & Pavlovic, 1995). All other bond lengths are within the expected ranges (Allen *et al.*, 1987). The crystal structure is stabilized by intermolecular O-H···N hydrogen bonds linking the neighbouring molecules into infinite chains along the *b* axis.

### Experimental

4-Bromo-4'-hydroxybenzylideneaniline was prepared by mixing equimolar amounts of 4-hydroxy benzaldehyde and 4-bromo aniline in ethanol (40 ml). The reaction mixture was refluxed for about 6 h and the resulting solution, kept at room temperature was slowly evaporated. After three days single crystals of the title compound, suitable for X-ray structure analysis were obtained.

### Refinement

All the H atoms were positioned geometrically and treated as riding on their parent atoms, with C-H = 0.93Å (aromatic), O–H = 0.82 Å and refined using a riding model with *U*_iso_(H)=1.2*U*_eq_(C) or 1.5*U*_eq_(O) for the hydroxy H atom.

### Crystal data

**Table:**

C_13_H_10_BrNO	*F*(000) = 1104
*M**_r_* = 276.13	*D*_x_ = 1.618 Mg m^−^^3^
Orthorhombic, *P**b**c**n*	Mo *K*α radiation, λ = 0.71073 Å
Hall symbol: -P 2n 2ab	Cell parameters from 6133 reflections
*a* = 21.9588 (10) Å	θ = 2.7–24.7°
*b* = 11.0866 (5) Å	µ = 3.60 mm^−^^1^
*c* = 9.3132 (4) Å	*T* = 293 K
*V* = 2267.28 (17) Å^3^	Block, brown
*Z* = 8	0.30 × 0.20 × 0.20 mm

### Data collection

**Table:**

Bruker Kappa APEXII CCD diffractometer	2001 independent reflections
Radiation source: fine-focus sealed tube	1494 reflections with *I* > 2σ(*I*)
Graphite monochromator	*R*_int_ = 0.046
ω and φ scans	θ_max_ = 25.0°, θ_min_ = 1.9°
Absorption correction: multi-scan (*SADABS*; Bruker, 2004)	*h* = −26→26
*T*_min_ = 0.452, *T*_max_ = 0.571	*k* = −12→13
20366 measured reflections	*l* = −8→11

### Refinement

**Table:**

Refinement on *F*^2^	Primary atom site location: structure-invariant direct methods
Least-squares matrix: full	Secondary atom site location: difference Fourier map
*R*[*F*^2^ > 2σ(*F*^2^)] = 0.056	Hydrogen site location: inferred from neighbouring sites
*wR*(*F*^2^) = 0.174	H-atom parameters constrained
*S* = 1.03	*w* = 1/[σ^2^(*F*_o_^2^) + (0.0898*P*)^2^ + 6.133*P*] where *P* = (*F*_o_^2^ + 2*F*_c_^2^)/3
2001 reflections	(Δ/σ)_max_ < 0.001
145 parameters	Δρ_max_ = 1.32 e Å^−^^3^
0 restraints	Δρ_min_ = −1.62 e Å^−^^3^

### Special details

**Table:**

Geometry. All e.s.d.'s (except the e.s.d. in the dihedral angle between two l.s. planes) are estimated using the full covariance matrix. The cell e.s.d.'s are taken into account individually in the estimation of e.s.d.'s in distances, angles and torsion angles; correlations between e.s.d.'s in cell parameters are only used when they are defined by crystal symmetry. An approximate (isotropic) treatment of cell e.s.d.'s is used for estimating e.s.d.'s involving l.s. planes.
Refinement. Refinement of *F*^2^ against ALL reflections. The weighted *R*-factor *wR* and goodness of fit *S* are based on *F*^2^, conventional *R*-factors *R* are based on *F*, with *F* set to zero for negative *F*^2^. The threshold expression of *F*^2^ > σ(*F*^2^) is used only for calculating *R*-factors(gt) *etc*. and is not relevant to the choice of reflections for refinement. *R*-factors based on *F*^2^ are statistically about twice as large as those based on *F*, and *R*- factors based on ALL data will be even larger.

### Fractional atomic coordinates and isotropic or equivalent isotropic displacement parameters (Å^2^)

**Table:**

	*x*	*y*	*z*	*U*_iso_*/*U*_eq_	
C1	0.3296 (2)	0.1888 (4)	−0.1451 (5)	0.0340 (10)	
C2	0.3501 (2)	0.0799 (4)	−0.0885 (5)	0.0398 (11)	
H2	0.3879	0.0493	−0.1156	0.048*	
C3	0.3145 (2)	0.0181 (4)	0.0074 (5)	0.0407 (11)	
H3	0.3283	−0.0551	0.0437	0.049*	
C4	0.2586 (2)	0.0620 (4)	0.0514 (5)	0.0333 (10)	
C5	0.2389 (2)	0.1715 (4)	−0.0048 (4)	0.0340 (10)	
H5	0.2014	0.2027	0.0231	0.041*	
C6	0.2743 (2)	0.2341 (4)	−0.1012 (5)	0.0340 (10)	
H6	0.2607	0.3076	−0.1370	0.041*	
C7	0.2200 (2)	−0.0109 (4)	0.1437 (5)	0.0360 (10)	
H7	0.2321	−0.0899	0.1619	0.043*	
C8	0.1337 (2)	−0.0598 (4)	0.2718 (5)	0.0342 (10)	
C9	0.1277 (2)	−0.1783 (5)	0.2249 (6)	0.0456 (12)	
H9	0.1487	−0.2041	0.1439	0.055*	
C10	0.0909 (3)	−0.2569 (5)	0.2975 (6)	0.0518 (13)	
H10	0.0870	−0.3359	0.2654	0.062*	
C11	0.0598 (2)	−0.2199 (5)	0.4172 (6)	0.0526 (14)	
C12	0.0639 (2)	−0.1040 (5)	0.4635 (6)	0.0518 (13)	
H12	0.0426	−0.0793	0.5445	0.062*	
C13	0.1002 (2)	−0.0229 (5)	0.3893 (5)	0.0450 (12)	
H13	0.1020	0.0571	0.4189	0.054*	
N1	0.17101 (17)	0.0253 (3)	0.2012 (4)	0.0342 (9)	
O1	0.36520 (16)	0.2436 (3)	−0.2426 (4)	0.0465 (9)	
H1	0.3522	0.3116	−0.2583	0.070*	
Br1	0.01108 (4)	−0.33090 (9)	0.51718 (9)	0.0918 (4)	

### Atomic displacement parameters (Å^2^)

**Table:**

	*U*^11^	*U*^22^	*U*^33^	*U*^12^	*U*^13^	*U*^23^
C1	0.034 (2)	0.032 (2)	0.036 (2)	−0.0054 (19)	0.0006 (19)	−0.0038 (19)
C2	0.032 (2)	0.037 (2)	0.050 (3)	−0.001 (2)	0.000 (2)	−0.003 (2)
C3	0.042 (3)	0.031 (2)	0.050 (3)	0.000 (2)	−0.006 (2)	0.005 (2)
C4	0.039 (2)	0.028 (2)	0.033 (2)	−0.0046 (19)	−0.0062 (19)	−0.0008 (19)
C5	0.036 (3)	0.032 (2)	0.034 (2)	−0.0006 (19)	−0.0022 (19)	−0.0030 (18)
C6	0.039 (3)	0.028 (2)	0.035 (2)	0.0001 (18)	−0.001 (2)	0.0015 (19)
C7	0.044 (3)	0.027 (2)	0.037 (3)	−0.003 (2)	−0.008 (2)	0.0033 (19)
C8	0.040 (3)	0.031 (2)	0.031 (2)	−0.0040 (19)	−0.0046 (19)	0.0030 (18)
C9	0.052 (3)	0.043 (3)	0.041 (3)	−0.010 (2)	0.000 (2)	−0.002 (2)
C10	0.057 (3)	0.041 (3)	0.058 (3)	−0.015 (2)	−0.008 (3)	0.005 (3)
C11	0.040 (3)	0.064 (4)	0.053 (3)	−0.014 (3)	−0.006 (2)	0.021 (3)
C12	0.040 (3)	0.069 (4)	0.047 (3)	0.002 (3)	0.006 (2)	0.004 (3)
C13	0.046 (3)	0.046 (3)	0.043 (3)	−0.001 (2)	0.000 (2)	−0.001 (2)
N1	0.041 (2)	0.0301 (19)	0.0314 (19)	−0.0041 (16)	−0.0036 (17)	0.0006 (16)
O1	0.046 (2)	0.0398 (19)	0.053 (2)	−0.0006 (15)	0.0120 (16)	0.0066 (16)
Br1	0.0712 (6)	0.1138 (7)	0.0904 (6)	−0.0442 (4)	0.0008 (4)	0.0423 (5)

### Geometric parameters (Å, º)

**Table:**

C1—O1	1.343 (5)	C8—C13	1.380 (7)
C1—C6	1.376 (6)	C8—C9	1.390 (7)
C1—C2	1.392 (6)	C8—N1	1.412 (6)
C2—C3	1.371 (7)	C9—C10	1.367 (7)
C2—H2	0.9300	C9—H9	0.9300
C3—C4	1.382 (7)	C10—C11	1.370 (9)
C3—H3	0.9300	C10—H10	0.9300
C4—C5	1.391 (6)	C11—C12	1.358 (8)
C4—C7	1.454 (6)	C11—Br1	1.878 (5)
C5—C6	1.375 (6)	C12—C13	1.386 (7)
C5—H5	0.9300	C12—H12	0.9300
C6—H6	0.9300	C13—H13	0.9300
C7—N1	1.267 (6)	O1—H1	0.8200
C7—H7	0.9300		
			
O1—C1—C6	123.4 (4)	C13—C8—C9	118.6 (4)
O1—C1—C2	117.4 (4)	C13—C8—N1	118.7 (4)
C6—C1—C2	119.3 (4)	C9—C8—N1	122.6 (4)
C3—C2—C1	119.7 (4)	C10—C9—C8	120.2 (5)
C3—C2—H2	120.2	C10—C9—H9	119.9
C1—C2—H2	120.2	C8—C9—H9	119.9
C2—C3—C4	121.6 (4)	C9—C10—C11	120.4 (5)
C2—C3—H3	119.2	C9—C10—H10	119.8
C4—C3—H3	119.2	C11—C10—H10	119.8
C3—C4—C5	118.2 (4)	C12—C11—C10	120.5 (5)
C3—C4—C7	119.8 (4)	C12—C11—Br1	120.0 (4)
C5—C4—C7	121.8 (4)	C10—C11—Br1	119.4 (4)
C6—C5—C4	120.6 (4)	C11—C12—C13	119.6 (5)
C6—C5—H5	119.7	C11—C12—H12	120.2
C4—C5—H5	119.7	C13—C12—H12	120.2
C5—C6—C1	120.6 (4)	C8—C13—C12	120.6 (5)
C5—C6—H6	119.7	C8—C13—H13	119.7
C1—C6—H6	119.7	C12—C13—H13	119.7
N1—C7—C4	124.7 (4)	C7—N1—C8	118.6 (4)
N1—C7—H7	117.7	C1—O1—H1	109.5
C4—C7—H7	117.7		
			
O1—C1—C2—C3	−177.6 (4)	N1—C8—C9—C10	179.9 (5)
C6—C1—C2—C3	1.5 (7)	C8—C9—C10—C11	−0.2 (8)
C1—C2—C3—C4	−0.9 (7)	C9—C10—C11—C12	1.5 (8)
C2—C3—C4—C5	0.2 (7)	C9—C10—C11—Br1	−178.6 (4)
C2—C3—C4—C7	174.8 (4)	C10—C11—C12—C13	−0.4 (8)
C3—C4—C5—C6	−0.1 (6)	Br1—C11—C12—C13	179.7 (4)
C7—C4—C5—C6	−174.6 (4)	C9—C8—C13—C12	3.4 (7)
C4—C5—C6—C1	0.7 (6)	N1—C8—C13—C12	−178.7 (4)
O1—C1—C6—C5	177.6 (4)	C11—C12—C13—C8	−2.2 (8)
C2—C1—C6—C5	−1.4 (7)	C4—C7—N1—C8	171.2 (4)
C3—C4—C7—N1	172.7 (4)	C13—C8—N1—C7	147.7 (4)
C5—C4—C7—N1	−12.9 (7)	C9—C8—N1—C7	−34.5 (6)
C13—C8—C9—C10	−2.2 (7)		

### Hydrogen-bond geometry (Å, º)

**Table:**

*D*—H···*A*	*D*—H	H···*A*	*D*···*A*	*D*—H···*A*
O1—H1···N1^i^	0.82	1.92	2.734	175

Symmetry code: (i) −*x*+1/2, −*y*+1/2, *z*−1/2.
